# Social and Physical Environmental Correlates of Adults’ Weekend Sitting Time and Moderating Effects of Retirement Status and Physical Health

**DOI:** 10.3390/ijerph110909790

**Published:** 2014-09-19

**Authors:** Veerle Van Holle, Sarah A. McNaughton, Megan Teychenne, Anna Timperio, Delfien Van Dyck, Ilse De Bourdeaudhuij, Jo Salmon

**Affiliations:** 1 Faculty of Medicine and Health Sciences, Department of Movement and Sport Sciences, Ghent University, Watersportlaan 2, 9000 Ghent, Belgium; E-Mails: delfien.vandyck@ugent.be (D.V.D.); ilse.debourdeaudhuij@ugent.be (I.D.B.); 2 Research Foundation Flanders (FWO), Egmontstraat 5, 1000 Brussels, Belgium; 3 Centre for Physical Activity and Nutrition Research, School of Exercise and Nutrition Sciences, Deakin University, Melbourne Burwood Campus, 221 Burwood Highway, Burwood VIC 3125, Australia; E-Mails: sarah.mcnaughton@deakin.edu.au (S.A.M.); megan.teychenne@deakin.edu.au (M.T.); anna.timperio@deakin.edu.au (A.T.); jo.salmon@deakin.edu.au (J.S.)

**Keywords:** sedentary behaviour, proximal social factors, social environment, physical environment, neighbourhood, retirement, health, ageing

## Abstract

Emerging research suggests that prolonged sedentary behaviour (SB) is detrimental to health. Changes in SB patterns are likely to occur during particular life stages, for example at retirement age (55–65-year-old). Evidence on socio-ecological SB correlates is scarce and inconsistent in this age group. Moreover, the influence of socio-ecological correlates may vary depending on health and retirement status. This study examined social and environment correlates of overall weekend day sitting among adults at or approaching retirement age, and moderating effects of perceived physical health and retirement status. Baseline data from the Wellbeing, Eating and Exercise for a Long Life study in 2839 Australian adults (55–65-year-old) were analysed. Participants self-reported proximal social factors, neighbourhood social and physical environment, physical health and retirement status. MLwiN multilevel regression analyses were conducted. In the multivariable model, only social support from friends/colleagues to discourage sitting (B = −0.891; *p* = 0.036) was associated with overall weekend day sitting. No moderation of retirement status, nor physical health were found in the multivariable results. Results from this study suggest the importance of social factors in relation to weekend day sitting among 55–65-year-old adults. Health promotion initiatives in this age group should pay special attention to enhancing social interaction opportunities. Moreover, findings suggest that SB-specific correlates may need to be examined in future research.

## 1. Introduction

It has been estimated that the global population of older adults will increase almost fourfold within the next four decades [1]. Since physical constraints, such as frailty and functional limitations typically increase with age [2], ageing will also put serious demands on the health care sector through the rise in institutionalised individuals. Therefore, it is crucial to target key behaviours detrimental to health and physical functioning, such as prolonged sedentariness [3], among individuals in pre-retirement and retirement in order to maintain older adults’ physical functioning and keep their independent community-based living as long as possible.

To date, public health research on energy balance-related behaviours has mainly focussed on the role of nutrition and physical activity (PA) in disease prevention [4,5]. However, sedentary behaviour (SB) or sitting may also pose an important independent risk to health. Research shows that many people who reach the public health recommendations for PA can still accumulate high daily levels of SB [6]. Moreover, there is emerging evidence of the negative health effects of SB (e.g., cardiovascular disease, metabolic syndrome, diabetes type II, cancers), independent of people’s moderate- to vigorous-intensity PA [7,8,9,10,11,12,13,14,15], which suggests the importance of developing health promotion programmes that specifically focus on reducing the time spent in SB, in addition to interventions targeting increases in PA levels.

Given that the volume of time spent in SB appears to increase with age [16], older adults may be particularly at risk of SB-related diseases. As with PA, SB occurs in various domains or lifestyle contexts, such as at home (primarily leisure-time), at work, or in transit (e.g., car) [17]. Furthermore, changes in PA are likely to occur during life events such as retirement [18]. Likewise, it is plausible that changes in SB are also likely to occur during particular life stages. People at the age of retirement (55–65-year-old), for instance, are in a phase where they experience a shift in the content of work-related and daily SBs [19] and are thus potentially a key target group for interventions influencing health behaviour. However, adequate knowledge of SB correlates is required in order to develop effective intervention programmes for people approaching retirement. Moreover, it is important to identify correlates of SB that have potential to be modified. Socio-ecological models to explain SB have been put forward during the past years. The socio-ecological framework described by Owen *et al.* [17] includes proximal factors (*i.e.*, intrapersonal factors; the perceived environment including social factors such as norms and support), as well as more distal factors (*i.e.*, behavioural settings such as home environment, neighbourhood social and physical environment; the policy environment) that are expected to explain adults’ SB. Potential SB correlates included in the present study were selected using this socio-ecological framework.

Retiring implies that occupational sitting will become less relevant for health promotion purposes. In contrast, sitting during discretionary time (leisure time) will constitute a larger part of retired adults’ SB and home and neighbourhood factors may become more important correlates of SB, because more time will be spent within the close vicinity of the home residence upon retirement. Health promotion initiatives may thus be most effective if they target reductions in the amount of discretionary SB adults at retirement age engage in. As the above-described socio-ecological correlates like the home and neighbourhood environment are most likely to play an important role in explaining discretionary, and not occupational sitting, it would be most relevant to examine the correlates of 55–65-year-olds’ leisure-time (discretionary) SB.

Although the literature regarding social correlates of PA among older adults is expanding, no studies focussing on social correlates of overall SB in this age group have been conducted so far. In adults aged 18–65-year-old, a small number of studies have primarily examined leisure-time SB such as television (TV) viewing, but these studies found inconsistent results regarding the importance of proximal social factors such as social support and social participation, and neighbourhood environmental factors such as social cohesion, interpersonal trust, and civic engagement. Specifically, proximal social environment factors and the neighbourhood social environment were negatively associated with TV time in Australian adult women [20]. In U.S. adults, similar negative relationships between neighbourhood social environmental factors and TV time were found [21], whereas another Australian study could not identify any association between social factors and adults’ TV viewing [22].

With regard to physical environmental correlates of SB, one study among older adults living in Canada examined urbanisation (metropolitan area) as a correlate of sitting time [23], but found no significant association. Among adults, recent research on physical environmental correlates of SB has focussed on outcome measures such as TV viewing, transport-related sitting and overall SB, but findings were inconsistent as well. Some studies found negative relationships between neighbourhood walkability, aesthetics-, and traffic-related factors and TV time [21,24,25]. In a three-country study, negative relationships were also observed between environmental activity-friendliness and motorized transport time in the total sample [26]. However, other research found no relationship between aspects of the physical environment and TV time [20] or overall sitting [25]. One study on overall SB levels even found a positive association with neighbourhood walkability [27].

A potential explanation for the inconsistent findings found in the literature to date, may be the scarcity of studies taking into account the moderating role of other potential influences on relationships between socio-environmental factors and SB. At retirement age, a key moderator of the relationship between socio-environmental factors and SB may be employment status. Ding *et al.* [22], for example, identified a significant interaction between walkability and retirement status on adults’ TV viewing time, with neighbourhood walkability being a negative correlate of TV viewing among unemployed participants only. The impact of employment status may be particularly important for people aged 55–65. In Australia, the average retirement age among recent retirees (last 5 years) was 61.4 years [28] and in 2010–2011, 42% of women and 11% of men aged 55–65 were not in the workforce or were retired [28]. These people are likely to spend a considerable amount of time (including leisure time) in their local neighbourhood as part of their daily routines. Compared with their retired peers, working adults may have sparser leisure time, most often limited to the weekend. Consequently, leisure activities of working adults, including engagement in SB, may be more consciously planned and could therefore be less dependent on social or physical environmental factors compared to those who are not in the workforce. So far, however, no studies have investigated moderating effects of employment status on relationships with SB during discretionary time such as weekend days in older adults.

Another factor that may influence associations between socio-environmental factors and SB is people’s perceived health status. Previous research has shown that positive associations exist between poorer self-rated health and TV viewing time [21]. Besides, perceived health status is associated with perceptions of a less favourable social and physical environment [29,30]. This might suggest that SB correlates could be different according to perceived health status.

In summary, social and physical environmental correlates of SB among adults are understudied and research focussing on people approaching or at retirement age is lacking, especially on SB during discretionary time such as on weekend days. Moreover, it is possible that correlates of SB in this group may vary according to retirement status or self-rated health profile. Increasing knowledge on modifiable correlates of SB in older adults is important for informing the development of targeted interventions to reduce SB, particularly among older adults most at risk. The current study aimed to investigate the relationship between several social and physical environmental aspects of the home and neighbourhood environments and weekend sitting time in adults approaching or at retirement age. A second aim was to investigate the moderating effects of retirement status and perceptions of self-rated physical health status on these relationships.

## 2. Methods

The present study utilised baseline data (2010) from the Wellbeing, Eating and Exercise for a Long Life (WELL) study. The WELL study was designed to investigate intrapersonal, social and physical environmental influences on nutrition and PA behaviours in Australian older adults (55–65-year-old). The WELL sampling strategy and study protocol have been previously described in detail [31,32]. Briefly, participants were recruited from 84 suburbs of Victoria, Australia, selected based on stratification by urban and rural area and area-level disadvantage. A random sample of 55–65-year-old residents within each suburb were mailed an invitation letter to participate in the WELL study and a postal questionnaire on personal, social and physical environmental influences on their dietary and PA behaviours. A total of 4082 participants returned completed surveys (38% response rate). All subjects gave their informed consent for inclusion before they participated in the study. The study was conducted in accordance with the Declaration of Helsinki, and the protocol was approved by the Human Research Ethical Committee of Deakin University (EC 2009-105).

### 2.1. Measures

#### 2.1.1. Demographics, Personal Variables and Weekly MVPA

Participants self-reported their age, sex, and highest education level (“no formal qualifications”, “year 10 or equivalent”, “year 12 or equivalent”, “trade/apprenticeship”, “certificate/diploma”, “university degree”, and “higher university degree”). For this study, three categories of education level were created: “did not complete high school”, “high school, trade/apprenticeship, certificate/diploma”, and “university or higher university degree”. Participants described their relationship status (“living in a registered marriage”, “living in a de facto relationship”, “separated”, “divorced”, “widowed”, or “never married”), which was recoded into two categories; “having a partner” and “having no partner”. Furthermore, participants reported the number of children (“none” (=0); “one” (=1); “two” (=2); “three” (=3); or “four or more” (=4)). The variable Body Mass Index (BMI) was derived from self-reported weight (kg) and height (cm) through the formula: BMI = (weight in kg /height in m^2^). Because PA levels are often negatively associated with SB levels, self-reported PA was assessed using the International Physical Activity Questionnaire long form (IPAQ-L, last 7 days version; [33]); a valid and reliable tool to assess PA and SB in adults [34,35]. Participants reported the number of days and the average time per day (hours and minutes) they were physically active in the last seven days. Hours were recoded as minutes and data were truncated at 1680 weekly minutes. Moreover, to diminish overestimations a correction factor of 0.80 was applied to this truncated variable. This yielded the variable “weekly MVPA”, expressed as minutes per week.

#### 2.1.2. Outcome Variable: Sitting Time during Weekend Days

Self-reported sitting time was also collected using IPAQ-L. Participants reported the average time (hours and minutes) they had spent sitting on weekdays and weekend days. Hours were recoded as minutes and data were truncated [36] at 960 min (~16 waking hours) and the minimum at 60 (~at least one hour of sitting, e.g., for having meals). As sitting on weekdays is largely influenced by occupation, only sitting during weekend days was used as an outcome variable in our study.

#### 2.1.3. Predictors

##### 2.1.3.1. Proximal Social Environment

“Social participation” was assessed from 13 items (Cronbach’s Alpha = 0.68), as described by [37]. Participants reported how often they had participated in the following activities during the past 12 months: “visited family or had family visit”; “visited friends or had friends visit”; “visited neighbours or had neighbours visit”; “been to a cafe or restaurant”; “been to a social club”; “been to the cinema or theatre”; “been to a party or dance”; “played sports”; “been to the gym or exercise class”; “been to a class (e.g., cooking, language)”; “been involved in a hobby group”; “been involved in a singing/acting/music group”; and “been involved in a self-help or support group”. Response categories for each item included: “not at all” (=1), “less than once per month” (=2), “about once or twice per month” (=3), or “more than twice per month” (=4). A mean score of these items was calculated.

“Social support from family” and “social support from friends or colleagues” were each assessed through a single-item question, adapted from previous items with reasonable test-retest reliability [38,39]: “During the past year, how often did members of your family discourage you from sitting around too much (e.g., watching too much TV)?” and “During the past year, how often did friends or work colleagues discourage you from sitting around too much (e.g., watching too much TV)?”, respectively. Possible responses were reported on a five-point scale: “never” (=1), “rarely” (=2), “a few times” (=3), “often” (=4) and “very often” (=5) (for both variables, higher scores corresponded with higher social support).

##### 2.1.3.2. Neighbourhood Social Environment

Participants were asked about “descriptive norms” in their neighbourhood: “I often see other people walking in my neighbourhood” and “I often see other people exercising (e.g., jogging, bicycling, playing sports) in my neighbourhood”. Responses were reported on a five-point Likert scale ranging from “strongly disagree” (=1) to “strongly agree” (=5) and a mean score was calculated (Cronbach’s Alpha = 0.81).

A measure of “social trust and cohesion”, adapted from Sampson [40], asked participants to report how much they agreed with the following statements on their local neighbourhood: “People in this neighbourhood can be trusted”; “This is a close-knit neighbourhood”; “People around here are willing to help their neighbours”; “People in this neighbourhood generally don’t get along with each other” (reverse coded); and “People in this neighbourhood do not share the same values” (reverse coded). Response options were the same as those used for the descriptive norms items. The mean of these five items was calculated (Cronbach’s Alpha = 0.79).

For “personal safety”, participants reported how much they agreed with the three statements: “I feel safe walking in my neighbourhood, day or night”; “Violence is not a problem in my neighbourhood”; and “My neighbourhood is safe from crime” [41]. Response options ranged from “strongly disagree” (=1) to “strongly agree” (=5). A mean of these three items was calculated (Cronbach’s Alpha = 0.79).

##### 2.1.3.3. Physical Environment

Six items were used to assess perceptions of “aesthetics” in the neighbourhood, based upon existing measures [41,42]. Participants reported their agreement with the following statements: “There is a lot of rubbish on the street in my neighbourhood” (reverse coded); “There is a lot of noise in my neighbourhood” (reverse coded); “In my neighbourhood the buildings and homes are well-maintained”; “The buildings and homes in my neighbourhood are interesting”; “My neighbourhood is attractive”; and “The trees in my neighbourhood provide enough shade”. Responses were recorded on a five-point Likert scale ranging from “strongly disagree” (=1) to “strongly agree” (=5). A mean score of all six items was calculated (Cronbach’s Alpha = 0.69).

A mean “destinations” score was computed based on three items adapted from Mujahid *et al.* [41]. Participants reported their agreement with the following statements on a five-point Likert scale ranging from “strongly disagree” (=1) to “strongly agree” (=5): “My neighbourhood offers many opportunities to be physically active”; “Local sports clubs and other facilities in my neighbourhood offer many opportunities to get exercise”; and “In my neighbourhood it is easy to walk places”. A mean score of these three items was calculated (Cronbach’s Alpha = 0.84). Participants were also asked to report the number of TV’s in the house, by choosing “none”, “one”, “two”, “three”, or “four or more”. As few participants chose “none” (0.6%) or “four or more” (8.3%), three groups were created (“≤1 TV”, “2 TV’s”, and “≥3 TV’s”).

#### 2.1.4. Moderators

Participants reported their employment status by describing their current main daily activities and/or responsibilities: “working full-time”; “working part-time”; “unemployed or laid off”; “keeping house and/or raising children full-time”; “studying full-time”; or “retired”. These options were collapsed to create the variable “retirement status”: “retired”; “working” (comprising working full-time, part-time, and studying). As participants who reported they were unemployed/laid off or keeping house/raising children full-time also tend to spend a large amount of their time in and around their houses, but cannot be considered “retired”, these participants were excluded from the analyses because they might have caused biasing of the results. Perceived physical health was assessed through the Short Form 36 item Survey (SF-36) [43]. Subscales on physical functioning (PF), role-physical function (RP), bodily pain (BP) and general perceptions of one’s physical health (GH) were calculated and a score /100 was derived according to the SF-36 scoring protocol [44]. All subscales showed very good internal consistency, with Cronbach’s Alpha of 0.89, 0.89, 0.87, and 0.80 for PF, RP, BP and GH, respectively. A mean score of all subscales was subsequently calculated, resulting in a total measure of “physical health status”. This measure was dichotomised at its median value, yielding the categories “poorer health” and “better health”.

### 2.2. Statistical Analyses

Prior to data analysis, missing cases in any of the aforementioned variables were excluded. This comprised missing cases in weekend day sitting/weekly levels of MVPA (4.3%); age (0.4%); retirement status (1.9%); relationship status (0.1%); educational level (1.9%); number of children (0.1%); BMI (4.2%); social participation (0.3%); social support from family (12.7%); social support from friends/colleagues (11.5%); descriptive norms (0.9%); social trust and cohesion (0.7%); personal safety (0.7%); aesthetics (0.8%); destinations (0.9%) and number of TVs (0.8%). Descriptive statistics of all included variables and preliminary analyses between groups (stratified by retirement status and physical health status) were conducted using SPSS, version 20.0 (SPSS Inc., Chicago, IL, USA). Because sitting time during weekend days was positively skewed, square root transformations were applied to improve normality in the data. Both items on social support (proximal social environment) were positively skewed and were logarithmically transformed. Transformed variables were used in all analyses reported below, except for their descriptive statistics, which were calculated with the raw data. MLwiN 2.25 multilevel linear regression modelling (two level: suburb-participant) was used to account for clustering of participants within areas of residence. Except for “number of TV’s”, all predictor variables were included in the analyses as continuous variables [45]. Associations between all independent variables and weekend day sitting, and moderating effects of retirement status and perceived physical health, respectively, were calculated in two consecutive steps. First, bivariate analyses were conducted to calculate main effects with sitting time during weekend days and first order interaction effects with perceived physical health and retirement status for each separate independent variable. If these initial analyses yielded a moderating effect of *p* < 0.10 [46], *post-hoc* linear regression models were conducted to investigate the associations between the independent variables and weekend sitting time within each category of the moderator (*i.e.*, retirement and physical health status). In a second step, main and interaction terms with *p* < 0.10 in the bivariate analyses were combined in a multivariable model. Furthermore, since weekend sitting time was significantly different by sex, educational level, relationship status, age at survey BMI and weekly MVPA, all above-mentioned analyses controlled for these variables. Statistical significance for the multivariable model was set at *p* < 0.05.

## 3. Results

### 3.1. Descriptive Statistics

Descriptive statistics of the sample and all included variables are presented in Table 1. In total, 2839 participants (48.4% women) with complete data were included for analysis, of which 47% were urban residents and 34% reported to be retired at the time of completing the survey. Mean age of the total sample was 60 ± 3 years and the retired group was significantly older than the non-retired group (62 ± 3 *vs.* 59 ± 3 years respectively; *p* < 0.001). Mean self-reported time spent sitting on weekdays was 382 ± 215 min per day (~6.4 h) and time spent sitting on weekends was 329 ± 167 min per day (~ 5.5 h). Preliminary analyses showed that sitting time during weekdays was significantly lower for those who were retired compared to those who were not (*p* < 0.001; data not shown). On the contrary, sitting time during weekend days was significantly higher for those who were retired compared to those who were not (*p* < 0.001; data not shown). Regarding perceived physical health, both for week- and weekend days, sitting time was higher for those with poorer perceived physical health compared to those with better perceived physical health (*p* = 0.001 for weekdays; *p* < 0.001 for weekend days, respectively; data not shown).

### 3.2. Bivariate and Multivariable Correlates of Weekend Day Sitting Time 

Table 2 presents the main effects of the bivariate analyses for the associations between each independent variable and participants’ weekend day sitting, as well as first order interaction effects with retirement status and physical health status. Furthermore, Table 2 displays results from the multivariable model, combining the independent variables and interaction terms with *p*-values <0.10 in the bivariate analyses. This multivariable model explained 9.1% of the total variance in sitting time on weekend days.

#### 3.2.1. Main Effects

##### 3.2.1.1. Proximal Social Environment Factors

Higher levels of “social participation” and greater perceived “social support for discouraging sitting from friends/colleagues” were associated with less sitting on weekends in the bivariate analyses (*p* = 0.076; *p* = 0.068, respectively). The main effect for social support from friends/colleagues also remained significant in the multivariable model (*p* = 0.036), whereas social participation was no longer associated with weekend sitting time in the multivariable model (*p* = 0.351). There was no significant bivariate relationship between “social support for discouraging sitting from family” and weekend sitting time (*p* = 0.239). Consequently, this variable was not included in the multivariable model.

**Table 1:** Descriptive statistics of included variables.

Variable	All	Retired	Working	Poorer Health	Better Health
	*n = 2839*	*n = 976*	*n = 1863*	*n = 1390*	*n = 1449*
Overall minutes of weekend day sitting					
M ± SD	329.2 ± 167.1	350.1 ± 171.5	318.2 ± 163.7	351.8 ± 176.9	307.5 ± 155.1
Median; interquartile range	300; 180	300; 180	300; 150	300; 180	270; 180
Overall minutes of weekday sitting					
M ± SD	382.5 ± 214.2	357.6 ± 195.2	395.5 ± 222.4	395.6 ± 215.2	370.0 ± 212.5
Median; interquartile range	300; 180	300; 180	360; 300	337.5; 180	300; 260
Age in years (M ± SD)	60.2 ± 3.2	61.8 ± 2.9	59.4 ± 3.0	60.4 ± 3.2	60.0 ± 3.1
Area of residence (%)					
Urban	47.1	40.6	50.5	43.0	51.0
Rural	52.9	59.4	49.5	57.0	49.0
Sex (% female)	48.4	55.7	44.5	47.2	49.5
Retirement status (% retired)	34.4			41.7	27.3
Physical health status (% poorer)	49.0	59.4	43.5		
Relationship status (% having a partner)	82.3	81.7	82.7	79.7	84.8
Number of children	2.2 ± 1.2	2.2 ± 1.2	2.2 ± 1.2	2.2 ± 1.2	2.2 ± 1.2
Level of education (%)					
Did not complete high school	34.3	41.3	30.6	38.8	30.0
High school, trade/apprenticeship, certificate/diploma	37.0	34.8	38.2	37.7	36.4
University or higher university degree	28.7	23.9	31.2	23.5	33.6
Body Mass Index in kg/m^2^ (M ± SD)	27.2 ± 4.7	27.6 ± 5.0	27.0 ± 4.5	28.3 ± 5.0	26.2 ± 4.0
Self-reported moderate-to-vigorous PA (min∙wk^−1^)					
M ± SD	903.2 ± 449.6	893.6 ± 442.4	908.2 ± 453.3	853.3 ± 465.1	951.1 ± 428.8
Median; interquartile range	1012; 852	986; 840	1032; 864	912; 924	1112; 768
Proximal social environment					
Social participation (/4; M ± SD)	2.1 ± 0.4	2.1 ± 0.4	2.1 ± 0.4	2.0 ± 0.4	2.1 ± 0.4
Social support family (/5; M ± SD)	2.0 ± 1.3	2.0 ± 1.3	1.9 ± 1.1	2.1 ± 1.3	1.9 ± 1.1
Social support friends/colleagues (/5; M ± SD)	1.4 ± 0.9	1.5 ± 0.9	1.4 ± 0.8	1.5 ± 0.9	1.4 ± 0.8
Neighbourhood social environment					
Descriptive norms (/5; M ± SD)	4.0 ± 0.7	4.0 ± 0.7	4.0 ± 0.7	3.9 ± 0.7	4.1 ± 0.7
Social trust and cohesion (:5; M ± SD)	3.6 ± 0.6	3.6 ± 0.6	3.5 ± 0.6	3.5 ± 0.6	3.6 ± 0.6
Personal safety (/5; M ± SD)	3.6 ± 0.7	3.6 ± 0.8	3.6 ± 0.7	3.5 ± 0.8	3.7 ± 0.7
Physical environment					
Neighbourhood aesthetics (/5; M ± SD)	3.8 ± 0.5	3.7 ± 0.5	3.8 ± 0.5	3.7 ± 0.5	3.8 ± 0.5
Neighbourhood destinations ((/5; M ± SD)	3.8 ± 0.7	3.8 ± 0.7	3.8 ± 0.7	3.7 ± 0.7	3.9 ± 0.6
N° of TV’s in the house					
One or less (%)	25.6	25.3	25.8	24.7	26.6
Two TV’s (%)	42.2	44.2	41.1	42.4	42.0
Three or more TV’s (%)	32.2	30.5	33.1	32.9	31.4

Notes: M = mean; SD = standard deviation.

**Table 2:** Bivariate and multivariate results for social and environmental correlates of weekend day sitting.

Main Effects	Bivariate Results ^1^	Multivariable Model ^2^
	B ± SE	B ± SE
Social participation	−0.340 ± 0.192 ^¥^	−0.186 ± 0.199
Social support family	0.393 ± 0.334	
Social support friends/colleagues	−0.774 ± 0.424 ^¥^	−0.891 ± 0.426 *
Descriptive norms	−0.085 ± 0.116	
Social trust and cohesion	−0.082 ± 0.172	−0.045 ± 0.193
Personal safety	0.009 ± 0.134	0.033 ± 0.148
Aesthetics	−0.390 ± 0.219 ^¥^	−0.184 ± 0.234
Destinations	−0.151 ± 0.122	
No. of TV’s in the house (ref. one or less)		
Two TV’s	−0.138 ± 0.199	
Three or more TV’s	0.101 ± 0.214	
Interaction Effects Retirement (ref. = not retired)	0.100 ± 0.380	
Social participation × retirement		
Social support family × retirement	−0.518 ± 0.664	
Social support friends/colleagues × retirement	−0.164 ± 0.857	
Descriptive norms × retirement	−0.050 ± 0.242	
Social trust and cohesion × retirement	−0.653 ± 0.290 *	−0.490 ± 0.315
ersonal safety × retirement	−0.478 ± 0.217 *	−0.268 ± 0.236
Aesthetics × retirement	0.059 ± 0.308	
Destinations × retirement	0.087 ± 0.249	
No. of TV’s in the house (ref. one or less)		
Two TV’s × retirement	−0.245 ± 0.410	
Three or more TV’s × retirement	0.008 ± 0.438	
Interaction Effects Physical Health (ref. = poorer)		
Social participation × physical health	−0.176 ± 0.373	
Social support family × physical health	−0.241 ± 0.646	
Social support friends/colleagues × physical health	−1.092 ± 0.844	
Descriptive norms × physical health	−0.080 ± 0.229	
Social trust and cohesion × physical health	0.113 ± 0.277	
Personal safety × physical health	0.185 ± 0.211	
Aesthetics × physical health	0.503 ± 0.302 ^¥^	0.383 ± 0.302
Destinations × physical health	0.178 ± 0.240	
No. of TV’s in the house (ref. one or less)		
Two TV’s × physical health	0.296 ± 0.391	
Three or more TV’s × physical health	−0.196 ± 0.413	

Notes: B = regression coefficient; SE = standard error; * *p* < 0.05; ^¥^ *p* < 0.10; ^1^ Adjusted for sex, education, relationship status, number of children, age, BMI and MVPA; ^2^ Adjusted for sex, education, relationship status, number of children, age, BMI, MVPA and all other predictors included in the model. The total model explained 9.1% of the total variance in overall minutes of weekend day sitting.

##### 3.2.1.2. Neighbourhood Social Environment

Neither neighbourhood “descriptive norms”, “social trust and cohesion”, nor “personal safety” were related to weekend day sitting in the bivariate analyses (*p* = 0.456; *p* = 0.635; *p* = 0.944, respectively). Consequently, these variables were not included in the multivariable model.

##### 3.2.1.3. Physical Environment

Higher “aesthetics” scores were associated with less time spent sitting on weekends in the bivariate analyses (*p* = 0.075), but this variable was no longer related to weekend sitting time in the multivariable model (*p* = 0.428). There was no significant bivariate relationship between “destinations”, “having 2 TV’s in the house” or “having 3 or more TV’s in the house”, and weekend sitting time (*p* = 0.216; *p* = 0.488; *p* = 0.637, respectively). Consequently, these variables were excluded from the multivariable model.

#### 3.2.2. Moderating Effects

##### 3.2.2.1. Retirement Status

**Figure 1:**
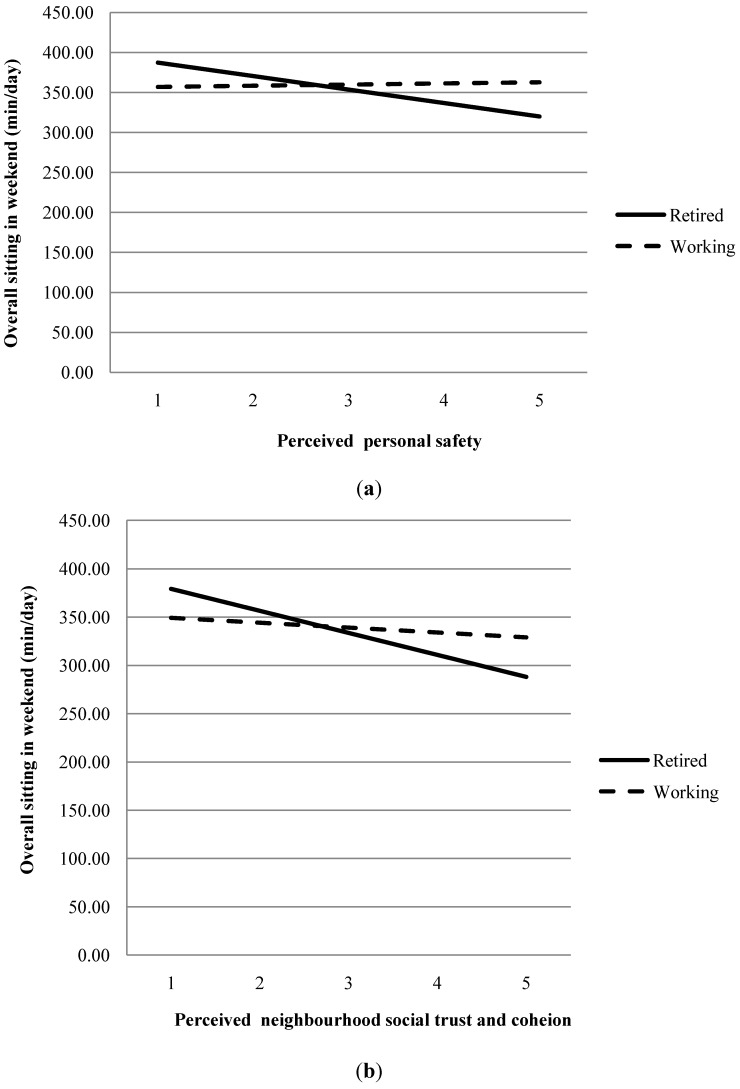
Moderation by retirement status for the bivariate relationship between social trust & cohesion (**a**), personal safety (**b**) and weekend day sitting.

Notes: Figure 1a shows a negative association between neighbourhood social trust and cohesion and overall sitting in weekend for retired adults (B = −0.581 ± 0.234; *p* = 0.013), whereas no association was found for working adults (B = −0.130 ± 0.175; *p* = 0.460). Likewise, Figure 1b shows a negative relationship between personal safety and overall sitting in weekend for retired adults (B = −0.405 ± 0.175; *p* = 0.020), but no association was found for working adults (B= 0.012 ± 0.136; *p* = 0.929).

Retirement status moderated the bivariate relationship between neighbourhood “social trust and cohesion” and weekend day sitting (*p* = 0.024), as well as the association between “personal safety” and weekend day sitting (*p* = 0.027). Both moderation effects are illustrated in Figure 1 and show that better perceptions of “neighbourhood social trust and cohesion” and “personal safety” were associated with less sitting time on weekends, but only in retired adults (*p* = 0.013; *p* = 0.020, respectively). There were no other moderation effects of retirement status on any of the bivariate relationships between the other independent variables and weekend day sitting, so these interaction terms were excluded from the multivariable analyses. In the multivariable model, retirement status no longer interacted with “social trust and cohesion” or “personal safety” (*p* = 0.120; *p* = 0.257, respectively).

##### 3.2.2.2. Perceived Physical Health Status

**Figure 2:**
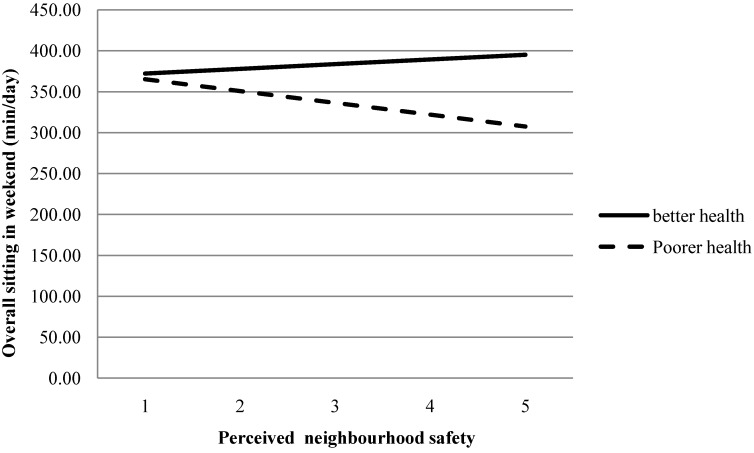
Moderation by health status for the bivariate relationship between neighbourhood aesthetics and weekend day sitting.

Notes: Figure 2 shows a negative association between aesthetics and overall sitting in weekend for adults with poorer health perceptions (B = −0.380 ± 0.228; *p* = 0.096), but no association was observed for adults with better health perceptions (B = −0.135 ± 0.205; *p* = 0.511).

In the bivariate analyses, perceived physical health status moderated the relationship between “neighbourhood aesthetics” and weekend sitting time (*p* = 0.095), which is shown in Figure 2. Specifically, higher “neighbourhood aesthetics” scores were associated with less weekend sitting only among participants with poorer health perceptions (*p* = 0.096). Perceived physical health status did not moderate any other bivariate relationship between the independent variables and weekend day sitting, so these interaction terms were excluded from the multivariable model. In the multivariable model, the interaction between physical health and “neighbourhood aesthetics” was no longer significant (*p* = 0.204).

## 4. Discussion

The present study aimed to investigate whether social and physical environmental factors were associated with sitting time on weekends in a sample of adults approaching or at retirement age; and to determine whether retirement status and physical health status moderated any associations between these variables. Research on socio-ecological correlates of SB in adults approaching or of retirement age is limited. Given that emerging evidence suggests that reducing prolonged sitting may be an important health promotion target and adults around retirement age are the ideal target population for implementing effective health promotion programs, the findings of our study are of high relevance within the public health domain.

Multivariable results of our study showed that only greater perceived social support from friends/colleagues to discourage sitting was associated with less overall minutes of weekend day sitting. Although on average, reports of social support were low, this could suggest that compared to none, even low amounts of support are related to less sitting time, which is a promising finding. Also PA-related research has identified social support as a positive predictor of health-enhancing and overall PA [47,48,49]. Moreover, our finding is consistent with one of the few studies that examined social factors as correlates of SB [20], where an inverse relationship was observed between Australian women’s TV viewing and social support from friends (in terms of encouragement to engage in PA). Regarding the measure of “social support from friends/colleagues”, a unique aspect of the present study is that participants were asked about social support to reduce sitting specifically, which is a very different concept to support for PA. To the best of our knowledge, no other SB-related research has examined social support from friends/colleagues to reduce sitting as a correlate of any sedentary outcome. Presumably, in the present study, higher levels of perceived social support have a similar health-enhancing effect as they have on increasing PA, but now within the context of lowering SB levels. The role of friends or colleagues in encouraging health behaviours may thus be important, and further research is required to better understand how to engage friends or colleagues to support individuals to reduce excessive sitting and be more physically active.

None of the neighbourhood social, nor physical environmental variables were significantly associated with weekend day sitting in the multivariable results of the current study, which contrasts with the findings of a study in U.S. adults, where measures of personal safety, as well as better aesthetics-related features were associated with less TV viewing [21]. However, our lack of associations between neighbourhood social and physical environmental factors and sitting time on weekends are consistent with a study on Belgian adults’ overall SB levels [26], and with above-mentioned Australian study on women’s TV viewing time [20]. Our results may suggest that among people around retirement age, neighbourhood social and physical environmental factors might be less important in explaining weekend sitting time, perhaps because they are less proximal influences on the behaviour and potentially only indirectly related to sitting during discretionary time. In contrast, factors such as social support are more likely to directly affect sitting behaviour because they are more individually-oriented. In addition, except for the social support measures, all predictors included in the analyses are known to be PA-related socio-ecological correlates, but their association with sitting behaviour remains inconclusive in the literature. For instance, it could be that the neighbourhood-related correlates are of less importance for SB, and SB may be determined by other factors such as the environment inside the home (more proximal environment) or by automatic processes or habit strength [50].

Although some moderating effects of retirement and physical health status were found in the bivariate analyses (*i.e.*, social trust and cohesion, personal safety, and aesthetics), none were observed in the multivariable results. This lack of moderation suggests that the main effects of social participation and social support from friends/colleagues are most important for predicting sitting time in this specific age group. This could also indicate that for people around retirement age, there may be no need to tailor health promotion campaigns according to whether or not someone has already retired or has a better *versus* poorer perceived physical health.

### Strengths and Limitations

This study was the first to investigate relationships between several social and physical environmental factors and SB in a specific population of adults approaching or at retirement age. Results were based on a large sample of community-dwelling participants, living in diverse geographic areas (*i.e.*, urban and rural regions; low, middle and high socio-economic index tertiles) across Victoria. Not only does this increase interpersonal variation within the study sample, it also enhances generalizability of the current findings. However, this generalizability might be limited because only weekend day sitting was used as an outcome variable. Other correlates may apply to weekday sitting during discretionary time and future studies should examine such correlates as well. A second limitation is the cross-sectional design where relationships cannot be assumed to be causal, thus current findings should be interpreted cautiously. Fortunately, WELL was designed to collect data across three time points [31] and future studies with this cohort could investigate temporal associations between the factors examined in the present study. For instance, longitudinal analyses will be able to examine how changes in working status are related to changes in SB and may add valuable knowledge to the small body of literature available [51,52]. Besides, not only retirement per se, but also increasing age and decreasing physical health should be taken into account when conducting longitudinal studies that look at the impact of retirement on SB in older adults. Thirdly, our outcome variable only focussed on weekend day sitting. Although this enhances comparability of sitting during discretionary time between working and retired adults, weekend day sitting has a short time frame and it would be interesting to look at SB correlates during both week and weekend days in future research. A fourth limitation is that our outcome variable was a global sitting measure, with specific domains such as sitting in a car not investigated. The relationship with social and physical environmental aspects of the ecological model for SB can vary across living contexts [17], with different correlates apparent for sitting in specific domains (e.g., transport-related sitting *vs.* occupational sitting; [25]. Our results showed that only a modest proportion of variance in overall weekend day sitting was explained by the multivariable model (9.1%). Perhaps, stronger associations may have been found if context-specific sitting, such as sitting at home, were investigated [53]. Future studies incorporating combinations of objective activity-monitors and previous-day reports of context-specific information [54,55] or GPS measurements may provide valuable information on these SB contexts. Also ecological momentary assessment is a useful method to collect context-specific information of SB [50]. Fifth, selection of potential SB correlates in the present study was based on predictors known to be related to PA. However, it could be that sitting time is partly determined by more habitual processes, according to what was observed in young adults [50]. Although such correlates of SB are not specifically included in the socio-ecological model that was used to frame the present study, they may be important determinants of SB, also in people at retirement age. Future studies are encouraged to incorporate both explicit and implicit/more automatic measures of potential SB correlates (e.g., measures related to habit strength). A further limitation is that only self-report data were collected. Previous methodological work has shown that participants tend to underestimate their time spent sitting [56]. Self-reported overall minutes of weekend day sitting time in our sample were very low on average (approximately 5.5 h), almost half as low as accelerometer-derived minutes of daily sedentary time (9.2 h) observed in another study amongst US adults aged ≥60-year-old [57]. Objective measurement methods such as inclinometers can provide more accurate data on the time spent sedentary or sitting, respectively [25,58]. Lastly, the reference periods used in the questionnaires were different (last 7 days *vs.* past 12 months) and could have obscured our findings.

## 5. Conclusions

In conclusion, this was the first study to investigate specific proximal social and neighbourhood environmental correlates of weekend day sitting in a unique population of adults approaching or at retirement age. Because in all age groups, there is still inconsistency regarding the most important correlates of sitting time, the present study adds to the existing knowledge in the field. Although bivariate results showed associations of weekend sitting time with proximal social factors, as well as with neighbourhood social and physical environmental predictors, only social support from friends/colleagues to discourage sitting remained statistically significantly related to weekend day sitting in the multivariable model. This suggests health promotion strategies targeting reduction of sitting time in adults aged between 55 and 65 may be most effective if they target direct influences on SB. Moreover, findings suggest that SB-specific correlates may need to be examined in future research. Additionally, no moderating effects of retirement status or physical health status were identified in the multivariable results of the present study, suggesting that for the specific group of people that are retired or about to enter retirement, there may be no need to tailor programmes promoting lower levels of sitting during discretionary time.

## References

[1] United Nations Department of Economic and Social Affairs World Population Ageing. http://www.un.org.

[2] Rockwood K., Howlett S.E., MacKnight C., Beattie B.L., Bergman H., Hébert R., Hogan D.B., Wolfson C., McDowell I. (2004). Prevalence, attributes, and outcomes of fitness and frailty in community-dwelling older adults: Report from the Canadian study of health and aging. J. Gerontol. Ser. A-Biol. Sci. Med..

[3] Santos D.A., Silva A.M., Baptista F., Santos R., Vale S., Mota J., Sardinha L.B. (2012). Sedentary behavior and physical activity are independently related to functional fitness in older adults. Exp. Gerontol..

[4] Kennedy E.T. (2006). Evidence for nutritional benefits in prolonging wellness. Amer. J. Clin. Nutr..

[5] Warburton D., Nicol C., Bredin S. (2006). Health benefits of physical activity: The evidence. Can. Med. Assoc. J..

[6] Owen N., Healy G.N., Matthews C.E., Dunstan D.W. (2010). Too much sitting: The population-health science of sedentary behavior. Exerc. Sport Sci. Rev..

[7] Katzmarzyk P.T., Church T.S., Craig C.L., Bouchard C. (2009). Sitting time and mortality from all causes, cardiovascular disease, and cancer. Med. Sci. Sport. Exerc..

[8] Bankoski A., Harris T.B., McClain J.J., Brychta R.J., Caserotti P., Chen K.Y., Berrigan D., Troiano R.P., Koster A. (2011). Sedentary activity associated with metabolic syndrome independent of physical activity. Diabetes Care.

[9] Gardiner P.A., Healy G.N., Eakin E.G., Clark B.K., Dunstan D.W., Shaw J.E., Zimmet P.Z., Owen N. (2011). Associations between television viewing time and overall sitting time with the metabolic syndrome in older men and women: The Australian diabetes, obesity and lifestyle study. J. Amer. Geriatr. Soc..

[10] Matthews C.E., George S.M., Moore S.C., Bowles H.R., Blair A., Park Y., Troiano R.P., Hollenbeck A., Schatzkin A. (2012). Amount of time spent in sedentary behaviors and cause-specific mortality in U.S. adults. Amer. J. Clin. Nutr..

[11] Thorp A.A., Owen N., Neuhaus M., Dunstan D.W. (2011). Sedentary behaviors and subsequent health outcomes in adults. A systematic review of longitudinal studies, 1996–2011. Amer. J. Prev. Med..

[12] Helmerhorst H.J.F., Wijndaele K., Brage S., Wareham N.J. (2009). Objectively measured sedentary time may predict insulin resistance independent of moderate- and vigorous-intensity physical activity. Diabetes.

[13] Krishnan S., Rosenberg L., Palmer J.R. (2009). Physical activity and television watching in relation to risk of type 2 diabetes: The black women’s health study. Amer. J. Epidemiol..

[14] Lynch B.M., Dunstan D.W., Vallance J.K., Owen N. (2013). Don’t take cancer sitting down: A new survivorship research agenda. Cancer.

[15] Ukawa S., Tamakoshi A., Wakai K., Kurozawa Y. (2014). Associations of daily walking and television viewing time with liver cancer mortality: Findings from the Japan collaborative cohort study. Cancer Causes Control.

[16] Ng S.W., Popkin B.M. (2012). Time use and physical activity: A shift away from movement across the globe. Obes. Rev..

[17] Owen N., Sugiyama T., Eakin E.E., Gardiner P.A., Tremblay M.S., Sallis J.F. (2011). Adults’ sedentary behavior determinants and interventions. Amer. J. Prev. Med..

[18] Allender S., Hutchinson L., Foster C. (2008). Life-change events and participation in physical activity: A systematic review. Health Promot. Int..

[19] Touvier M., Bertrais S., Charreire H., Vergnaud A.-C., Hercberg S., Oppert J.-M. (2010). Changes in leisure-time physical activity and sedentary behaviour at retirement: A prospective study in middle-aged French subjects. Int. J. Behav. Nutr. Phys. Act..

[20] Teychenne M., Ball K., Salmon J. (2012). Correlates of socio-economic inequalities in women’s television viewing: A study of intrapersonal, social and environmental mediators. Int. J. Behav. Nutr. Phys. Act..

[21] King A.C., Goldberg J.H., Salmon J., Owen N., Dunstan D., Weber D., Doyle C., Robinson T.N. (2010). Identifying subgroups of U.S. adults at risk for prolonged television viewing to inform program development. Amer. J. Prev. Med..

[22] Ding D., Sugiyama T., Winkler E., Cerin E., Wijndaele K., Owen N. (2012). Correlates of change in adults’ television viewing time: A four-year follow-up study. Med. Sci. Sport. Exerc..

[23] Dogra S., Stathokostas L. (2014). Correlates of extended sitting time in older adults: An exploratory cross-sectional analysis of the Canadian community health survey healthy aging cycle. Int. J. Public Health.

[24] Sugiyama T., Salmon J., Dunstan D.W., Bauman A.E., Owen N. (2007). Neighborhood walkability and TV viewing time among Australian adults. Amer. J. Prev. Med..

[25] Kozo J., Sallis J.F., Conway T.L., Kerr J., Cain K., Saelens B.E., Frank L.D., Owen N. (2012). Sedentary behaviors of adults in relation to neighborhood walkability and income. Health Psychol..

[26] Van Dyck D., Cerin E., Conway T.L., De Bourdeaudhuij I., Owen N., Kerr J., Cardon G., Frank L.D., Saelens B.E., Sallis J.F. (2012). Associations between perceived neighborhood environmental attributes and adults’ sedentary behavior: Findings from the USA., Australia and Belgium. Soc. Sci. Med..

[27] Van Dyck D., Cardon G., Deforche B., Owen N., Sallis J.F., De Bourdeaudhuij I. (2010). Neighborhood walkability and sedentary time in Belgian adults. Amer. J. Prev. Med..

[28] (2011). Retirement and Retirement Intentions Australia.

[29] Stafford M., Cummins S., Ellaway A., Sacker A., Wiggins R.D., Macintyre S. (2007). Pathways to obesity: Identifying local, modifiable determinants of physical activity and diet. Soc. Sci. Med..

[30] Yang L., Sahlqvist S., McMinn A., Griffin S.J., Ogilvie D. (2010). Interventions to promote cycling: Systematic review. Brit. Med. J..

[31] McNaughton S.A., Crawford D., Ball K., Salmon J. (2012). Understanding determinants of nutrition, physical activity and quality of life among older adults: The Wellbeing , Eating and Exercise for a Long Life (WELL ) study. Health Qual. Life Outcomes.

[32] Södergren M., McNaughton S.A., Salmon J., Ball K., Crawford D. (2012). Associations between fruit and vegetable intake, leisure-time physical activity, sitting time and self-rated health among older adults: Cross-sectional data from the WELL study. BMC Public Health.

[33] The International Physical Activity Questionnaire (IPAQ). http://www.ipaq.ki.se/.

[34] Craig C.L., Marshall A.L., Sjostrom M., Bauman A.E., Booth M.L., Ainsworth B.E., Pratt M., Ekelund U., Yngve A., Sallis J.F., Oja P. (2003). International physical activity questionnaire: 12-country reliability and validity. Med. Sci. Sport. Exerc..

[35] Bauman A., Ainsworth B.E., Sallis J.F., Hagströmer M., Craig C.L., Bull F.C., Pratt M., Venugopal K., Chau J., Sjöström M. (2011). The descriptive epidemiology of sitting. A 20-country comparison using the International Physical Activity Questionnaire (IPAQ). Amer. J. Prev. Med..

[36] Guidelines for Data Processing and Analysis of The International Physical Activity Questionnaire (IPAQ)—Short and Long Forms. http://www.ipaq.ki.se/scoring.pdf.

[37] Baum F.E., Bush R.A, Modra C.C., Murray C.J., Cox E.M., Alexander K.M., Potter R.C. (2000). Epidemiology of participation: An Australian community study. J. Epidemiol. Community Health.

[38] Ball K., Cleland V., Salmon J., Timperio A.F., McNaughton S., Thornton L., Campbell K., Jackson M., Baur L.A., Mishra G. (2013). Cohort profile: The Resilience for Eating and Activity Despite Inequality (READI) study. Int. J. Epidemiol..

[39] Sallis J.F., Grossman R.M., Pinski R.B., Patterson T.L., Nader P.R. (1987). The development of scales to measure social support for diet and exercise behaviors. Prev. Med..

[40] Sampson R.J., Raudenbush S.W., Earls F. (1997). Neighborhoods and violent crime: A multilevel study of collective efficacy. Science.

[41] Mujahid M.S., Diez Roux A.V, Morenoff J.D., Raghunathan T. (2007). Assessing the measurement properties of neighborhood scales: From psychometrics to ecometrics. Amer. J. Epidemiol..

[42] Ball K., Abbott G., Cleland V., Timperio A., Thornton L., Mishra G., Jeffery R.W., Brug J., King A., Crawford D. (2012). Resilience to obesity among socioeconomically disadvantaged women: The READI study. Int. J. Obes..

[43] Ware J.E., Sherbourne C.D. (1992). The MOS 36-Item Short-Form health survey (SF-36): I. Conceptual framework and item selection. Med. Care.

[44] 36-Item Short Form Survey from The RAND Medical Outcomes Study. http://www.rand.org/health/surveys_tools/mos/mos_core_36item.html.

[45] Nunnaly J.C., Bernstein I.H. (1994). Psychometric Theory.

[46] Twisk J.W.R. (2006). Applied Multilevel Analysis.

[47] Ball K., Timperio A., Salmon J., Giles-Corti B., Roberts R., Crawford D. (2007). Personal, social and environmental determinants of educational inequalities in walking: A multilevel study. J. Epidemiol. Community Health.

[48] Da Silva M., Crochemore I., Azevedo M.R., Goncalves H. (2013). Leisure-time physical activity and social support among Brazilian adults. J. Phys. Act. Health.

[49] Trost S.G., Owen N., Bauman A.E., Sallis J.F., Brown W. (2002). Correlates of adults’ participation in physical activity: Review and update. Med. Sci. Sport Exerc..

[50] Conroy D.E., Maher J.P., Elavsky S., Hyde A.L., Doerksen S.E. (2013). Sedentary behavior as a daily process regulated by habits and intentions. Heal. Psychol..

[51] Evenson K.R., Rosamond W.D., Cai J., Diez-Roux A.V., Brancati F.L. (2002). Influence of retirement on leisure-time physical activity: The atherosclerosis risk in communities study. Amer. J. Epidemiol..

[52] Clark B.K., Peeters G.M.E.E., Gomersall S.R., Pavey T.G., Brown W.J. (2014). Nine year changes in sitting time in young and mid-aged Australian women: Findings from the Australian longitudinal study for women’s health. Prev. Med..

[53] Giles-Corti B., Timperio A., Bull F., Pikora T. (2005). Understanding physical activity environmental correlates: Increased specificity for ecological models. Exerc. Sport Sci. Rev..

[54] Gomersall S.R., Olds T.S., Ridley K. (2011). Development and evaluation of an adult use-of-time instrument with an energy expenditure focus. J. Sci. Med. Sport.

[55] Kozey Keadle S., Lyden K., Hickey A., Ray E.L., Fowke J.H., Freedson P.S., Matthews C.E. (2014). Validation of a previous day recall for measuring the location and purpose of active and sedentary behaviors compared to direct observation. Int. J. Behav. Nutr. Phys. Act..

[56] Matton L., Wijndaele K., Duvigneaud N., Duquet W., Philippaerts R., Thomis M., Lefevre J. (2007). Reliability and validity of the flemish physical activity computerized questionnaire in adults. Res. Quart. Exercise Sport.

[57] Clark B.K., Healy G.N., Winkler E.A.H., Gardiner P.A., Sugiyama T., Dunstan D.W., Matthews C.E., Owen N. (2011). Relationship of television time with accelerometer-derived sedentary time: NHANES. Med. Sci. Sports Exerc..

[58] Jovanovic J.L., Hughes D.C., Baum G.P., Carmack C., Greisinger A.J., Basen-Engquist K. (2011). Accelerometry and self-report in sedentary populations. Amer. J. Health Behav..

